# Physiological Responses to the Foliar Application of Synthetic Resistance Elicitors in Cape Gooseberry Seedlings Infected with *Fusarium oxysporum* f. sp. *physali*

**DOI:** 10.3390/plants9020176

**Published:** 2020-02-01

**Authors:** Cristhian C. Chávez-Arias, Sandra Gómez-Caro, Hermann Restrepo-Díaz

**Affiliations:** Departamento de Agronomía, Facultad de Ciencias Agrarias, Universidad Nacional de Colombia, Carrera 30 No. 45-03, Bogotá 111321, Colombia; ccchaveza@unal.edu.co (C.C.C.-A.); sgomezc@unal.edu.co (S.G.-C.)

**Keywords:** foliar sprays, lipid peroxidation, plant defense mechanisms, rapid light-response curves, vascular wilt

## Abstract

Vascular wilt caused by *Fusarium oxysporum* is the most limiting disease that affects cape gooseberry (*Physalis peruviana* L.) crops in Colombia. The use of synthetic elicitors for vascular wilt management is still scarce in Andean fruit species. The objective of the present study was to evaluate the effect and number of foliar applications of synthetic elicitors such as jasmonic acid (JA), salicylic acid (SA), brassinosteroids (BR), or a commercial resistance elicitor based on botanical extracts (BE) on disease progress and their effect on the physiology of cape gooseberry plants inoculated with *F. oxysporum* f. sp. *physali*. Groups of ten plants were separately sprayed once, twice, or three times with a foliar synthetic elicitor, respectively. Elicitor applications were performed at the following concentrations: JA (10 mL L^−1^), SA (100 mg L^−1^), BR (1 mL L^−1^) and BE (2.5 mL of commercial product (Loker^®^) L^−1^). The results showed that three foliar BR, SA, or BE applications reduced the area under the disease progress, severity index, and vascular browning in comparison to inoculated plants without any elicitor spray. Three BR, SA, or BE sprays also favored stomatal conductance, water potential, growth (total dry weight and leaf area) and fluorescence parameters of chlorophyll compared with inoculated and untreated plants with no elicitor sprays. Three foliar sprays of SA, BR, or BE enhanced photosynthetic pigments (leaf total chlorophyll and carotenoid content) and proline synthesis and decreased oxidative stress in Foph-inoculated plants. In addition, the effectiveness of three foliar BR, SA, or BE sprays was corroborated by three-dimensional plot and biplot analysis, in which it can evidence that stomatal conductance, proline synthesis, and efficacy percentage were accurate parameters to predict Foph management. On the hand, JA showed the lowest level of amelioration of the negative effects of Foph inoculation. In conclusion, the use of the synthetic elicitors BR, SA, or BE can be considered as a tool complementary for the commercial management of vascular wilt in areas where this disease is a limiting factor.

## 1. Introduction

Cape gooseberry (*Physalis peruviana* L.) is a species that belongs to the Solanaceae family, native to the Peruvian Andean zones [1]. This species benefits human health due to its high vitamin, mineral (phosphorus and iron), and fiber contents, as well as its levels of antioxidant compounds (ascorbic acid and provitamin A) [2,3,4,5]. In Colombia, cape gooseberry crops occupied an area of 1023 ha with a production of 15,112 t in 2017 [6]. This species is also the second most exported fruit in the country followed by plantain and banana [1].

A reduction of cape gooseberry productivity has been registered (from 18 t/ha in 2009 to 15 t/ha in 2014) in the last years in Colombia [6,7]. One of the main causes of this reduction is vascular wilt, caused by the soil pathogen *Fusarium oxysporum* f. sp. *physali* (Foph) [8,9], which causes turgor loss in young leaves and stems, chlorosis in old leaves, branch drying and plant growth inhibition [8].

Chlamydospores production guarantees Foph long-term survival in the soil, making disease management difficult [7]. Vascular wilt control has been focused exclusively on the use of chemical fungicides, generating resistance by the pathogen, environmental contamination and low crop profitability [8,10]. Also, the implementation of cultural practices such as elimination of infected plants or soil solarization is scarce by growers [8]. This lack of control strategies has generated, in some cases, total crop death [9]. For this reason, the development of alternative methods for the management of soil pathogens has been of growing interest in recent years [11]. 

One of the strategies to mitigate the negative impact of plant pathogens on growth and crop yield is the application of synthetic elicitors which enhances plant defense mechanisms [12,13]. Elicitors are stable molecules that signal plant immune defense responses and improve plant physiological status under biotic stresses [14,15]. Different types of synthetic elicitors such as inorganic compounds, lipids, glycopeptides, and glycoproteins have been characterized and studied [13,16]. The most commonly studied synthetic elicitors against biotic stress are salicylic acid (SA) [17,18], jasmonic acid (JA) [19,20] and brassinosteroids (BR) [21,22].

SA plays a critical role in plant defense to biotic and abiotic stress [23,24]. Systemic acquired resistance (SAR) is one of the most representative forms of plant defense mechanisms induced by SA [17]. Foliar SA application has been used to promote SAR in pathogen-infected plants [20,25]. Also, exogenous SA application decreased leaf lesions caused by *Glomerella cingulata* and increased the total antioxidant capacity (catalase, superoxide dismutase and peroxidase) in apple (*Malus domestica* Borkh.) plants [26].

BR are involved in a large number of processes in plants, which mainly include seed germination, stem elongation, cell division and expansion, xylem differentiation and apical dominance [21]. Likewise, BR enhance metabolite synthesis (γ-Aminobutyric acid, D-galactose, and proline) and antioxidant machinery (peroxidase, catalase and ascorbate peroxidase) to protect plant cells from pathogen infestation [27,28,29]. Furio et al. [30] observed that foliar BR application induced a defense response in strawberry (*Fragaria x ananassaa*) plants infected with *Colletotrichum acutatum* by increasing lignin and callose deposits. Similarly, Canales et al. [31] showed that BR can be a useful tool for the management of Huanglongbing (HLB) in citrus trees (*Citrus aurantifolia* (Christm) Swingle).

JA is also a plant hormone that can promote plant tolerance against diseases by the induced systemic resistance (ISR) mechanism [32]. ISR promotes physiological processes that allow plants to react more efficiently to biotic stress [33,34]. Ávila et al. [35] found that foliar JA application prior to *Fusarium oxysporum* f.sp. *quitoense* inoculation protected lulo (*Solanum quitoense* L.) plants and generated the activation of the signaling pathway to induce resistance.

Commercial botanical extracts (BE) have been used to improve the plant’s immune system as a management strategy to reduce synthetic fungicides use [36,37]. The benefit of the application of these compounds is that they are highly biodegradable and toxic to several arthropod pests and pathogens [15,38,39]. Phenylpropanoids are aromatic hydrocarbons that constitute different botanical extracts and are involved in various disease resistance responses through secondary metabolites synthesis such as phenolics, flavonoids, lignin and anthocyanins [40]. In this sense, commercial products based on different botanical extracts are also rich in polysaccharides, and amino acids that can act as defense inducers in plants [41,42].

The physiological and biochemical plant responses are important traits to characterize possible defense mechanisms to biotic stress conditions associated with plant pathogens [43,44]. Physiological parameters such as leaf gas exchange properties, leaf water potential, and plant canopy growth and temperature have been used to characterize genotypes tolerance to pathogen infection [45]. Also, proline content has been correlated with plant defense against diseases [46]. In addition, malondialdehyde (MDA) production has also been used as a biochemical parameter to characterize plant responses to pathogens attack [44].

SA, JA, and BR are molecules that are involved in several plant physiological processes [47,48,49]. For example, SA is involved in leaf gas exchange (photosynthesis rate, stomatal conductance), water relations, and modulates plant response to environmental stress [50]. In turn, JA promotes several mechanisms to stress acclimation such as plant growth, morphological changes and proline accumulation [51,52]. Finally, BR can regulate stomatal closure and leaf senescence, and favor plant resistance to stress agents [53,54].

Studies on the use of synthetic elicitors have been mainly focused on the control of diseases through the induction of resistance mechanisms [17,19,21]. However, the available literature about elicitors use in disease management of Andean fruit trees such as cape gooseberry is still scarce. For this reason, the objective of the present study was to evaluate the effect and number of foliar applications of four different synthetic elicitors (salicylic acid (SA), brassinosteroids (BR), jasmonic acid (JA) and a commercial product (Loker^®^, Bologna, Italy) based on botanical extracts (BE)) on stomatal conductance, leaf water potential, chlorophyll fluorescence parameters, plant growth and biochemical responses of cape gooseberry seedlings infected with Foph.

## 2. Results

### 2.1. Vascular Wilt Severity Expressed as AUDPC, Disease Index, and Vascular Browning

**Figure 1:**
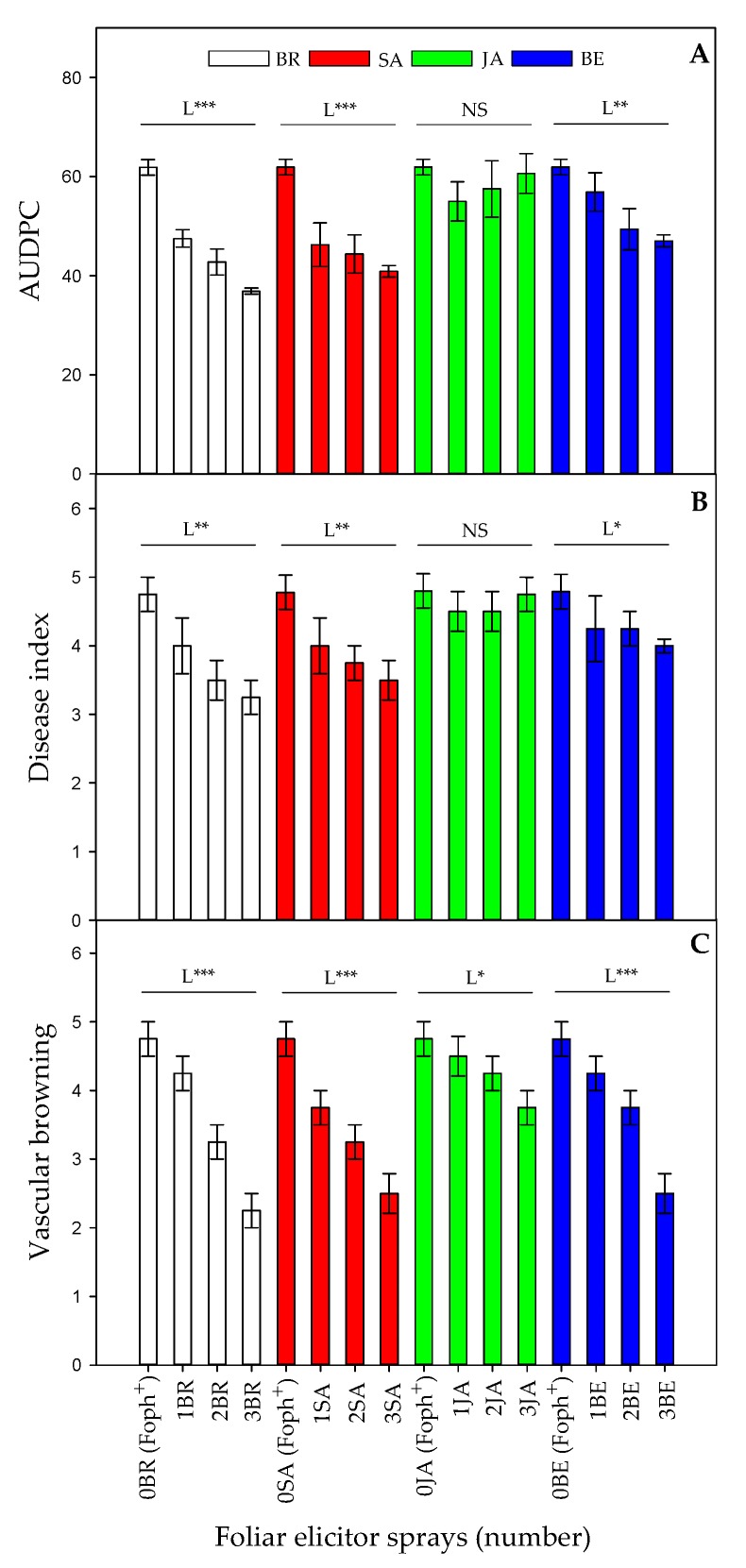
Effect of the interaction between synthetic elicitors (Salicylic acid (SA), jasmonic acid (JA), Brassinosteroids (BR), or botanical extracts (BE)) and the sprays number (0, 1, 2 and 3 applications) on area under the disease progress curve (AUDPC) (**A**), vascular wilt index (**B**) and vascular browning (**C**) in *Fusarium oxysporum* f. sp. *physali* (Foph^+^) infected cape gooseberry (*Physalis peruviana* L.) plants at 50 DAI. Each point represents the average of ten plants (*n* = 10). NS, not significant at α = 0.05. *, ** and *** significant at P≤ 0.05, 0.01 and 0.001, respectively. L = linear.

The area under the disease progress curve (AUDPC), the disease index and vascular browning are presented in Figure 1. Foph-inoculated plants from all treatments showed common symptoms of vascular wilt, and Foph presence was confirmed by isolates from affected material in PDA (data not shown). Also, this technique confirmed the pathogen’s absence in plants without inoculation and foliar elicitors applications (control plants).

Significant differences (*p* = 0.021) were observed in the interaction between factors on AUDPC, disease index and vascular browning. In general, a negative linear trend was observed in these variables (AUDPC, severity index and vascular browning) when number of foliar elicitor applications increased (Figure 1A–C). Three foliar BR or SA applications showed a lower vascular wilt AUDPC (36.8 and 40.8) and severity index (3.25 and 3.5) compared to BE (47 for AUDPC and 4 for the disease index), respectively (Figure 1A, B). On the other hand, plants treated with JA did not show any statistical differences in these two parameters compared with untreated inoculated plants. Regarding vascular browning percentage, a negative linear trend was observed between this variable and the foliar sprays number in all elicitors. Foph-inoculated plants and sprayed with BR (2.25), SA (2.5) and BE (2.5) showed lower vascular browning (Figure 1C). 

**Figure 2:**
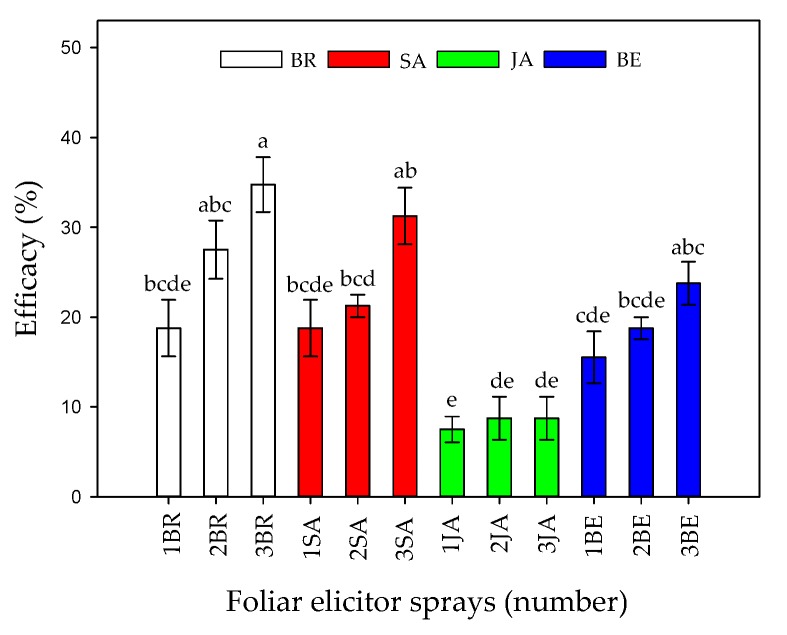
Effect of the interaction between synthetic elicitors (Salicylic acid (SA), jasmonic acid (JA), Brassinosteroids (BR), or botanical extracts (BE)) and the sprays number (0, 1, 2 and 3 applications) on control efficacy in *Fusarium oxysporum* f. sp. *physali* infected cape gooseberry (*Physalis peruviana* L.) plants at 50 DAI. Data represent the average of ten plants ± SE per treatment (*n* = 10). Bars followed by different letters indicate statistically significant differences according to the Tukey test (*p* ≤ 0.05).

**Figure 3:**
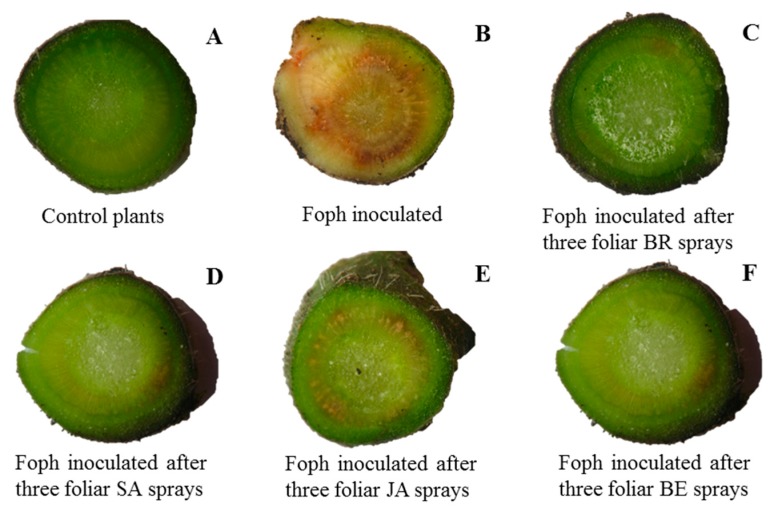
Vascular browning of cape gooseberry plants with different *Fusarium oxysporum* f. sp. *physali* (Foph^+^) inoculation and synthetic elicitors sprays. Control plants (non-inoculated plants and without any synthetic elicitor spray) (**A**), pathogen control plants (plants inoculated with only Foph) (**B**), Foph-inoculated plants with three foliar brassinosteroids (BR) applications (**C**), Foph-inoculated plants with three foliar salicylic acid (SA) applications (**D**), Foph-inoculated plants with three foliar jasmonic acid (JA) applications (**E**), and Foph-inoculated plants with three foliar botanical extracts (BE) applications (**F**) at 50 DAI.

Finally, the efficacy percentage showed that an increase in the number of foliar sprays, mainly BR, SA, or BE, was able to reduce symptoms in Foph-infected plants (Figure 2). The previous tendency is confirmed by Figure 3 in which the image shows that Foph-inoculated plants without any foliar elicitor spray had the greater vascular browning in comparison with the other treatments.

### 2.2. Plant Growth Variables

**Figure 4:**
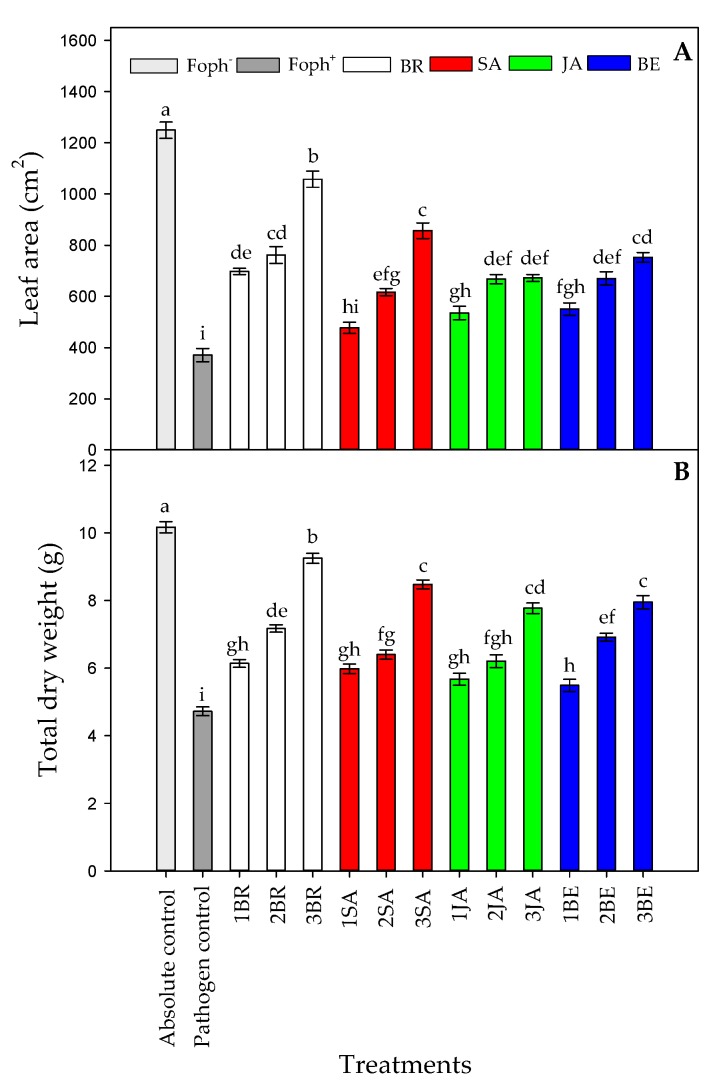
Effect of the different number of sprays (0, 1, 2 and 3 applications) of synthetic elicitors (Salicylic acid (SA), jasmonic acid (JA), Brassinosteroids (BR), or botanical extracts (BE)) on leaf area (LA) (**A**) and total dry weight (TDW) (**B**) in *Fusarium oxysporum* f. sp. *physali* (Foph^+^) infected cape gooseberry (*Physalis peruviana* L.) plants at 50 DAI. Data represent the average of ten plants ±SE per treatment (*n* = 10). Bars followed by different letters indicate statistically significant differences according to the Tukey test (*p* ≤ 0.05).

Significant differences were found between treatments (elicitors and foliar applications number) on leaf area (LA) (*p* = 0.000) and total plant dry weight (TDW) (*p* = 0.000) at 50 DAI. LA and TDW were highly affected by Foph inoculation, recording a lower value in this group of plants (pathogen control). When cape gooseberry plants were sprayed with elicitors, a positive response were observed. In this sense, three BR sprays caused a higher TDW (9.25 g) and LA (1057.4 cm^2^) values followed by SA sprayed plants three times (8.47 g for TDW and 856.7 cm^2^ for LA, respectively) (Figure 4A,B). On the other hand, Foph-inoculated plants treated with JA or BE did not show a significant increase in these growth parameter values at the end of the experiment. Finally, the highest LA (1259.3 cm^2^) and TDW (10.16 g) were recorded in the absolute (uninfected plants) control (Figure 4).

### 2.3. Stomatal Conductance (g_s_), Leaf Temperature (T_L_) and Leaf Water Potential (Ψ_wf_)

**Figure 5:**
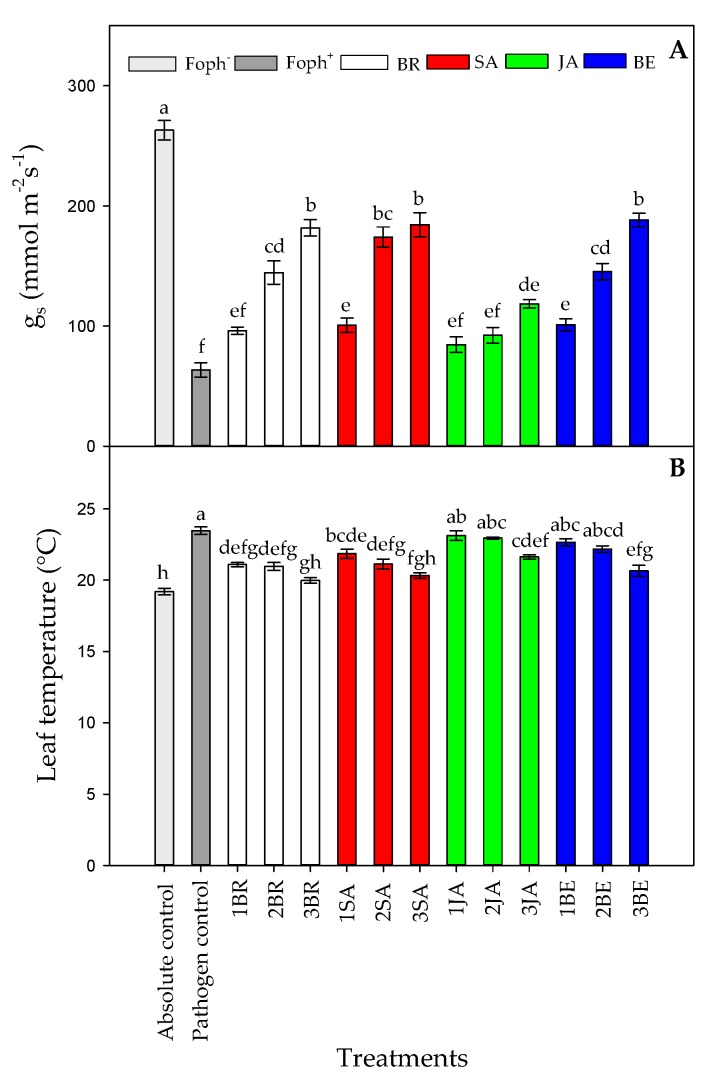
Effect of the different number of sprays (0, 1, 2 and 3 applications) of synthetic elicitors (Salicylic acid (SA), jasmonic acid (JA), Brassinosteroids (BR), or botanical extracts (BE)) on stomatal conductance (g_s_) (**A**) and leaf temperature (T_L_) (**B**) in *Fusarium oxysporum* f. sp. *physali* (Foph^+^) infected cape gooseberry (*Physalis peruviana* L.) plants at 50 DAI. Data represent the average of ten plants ±SE per treatment (*n* = 10). Bars followed by different letters indicate statistically significant differences according to the Tukey test (*p* ≤ 0.05).

Statistical differences were also found between treatments (synthetic elicitors and foliar sprays number) on g_s_ (*p* = 0.000), T_L_ (*p* = 0.001), and Ψ_wf_ (*p* = 0.000) at 50 DAI. Uninfected plants and not treated with any elicitor (absolute control) had the highest values in g_s_ (263.01 mmol m^−2^ s^−1^) (Figure 5A). 

In contrast, Foph plants showed the lowest gs (63.58 mmol m^−2^ s^−1^). On the other hand, foliar elicitor sprays helped to increase this variable under inoculate conditions. In general, three foliar synthetic elicitor applications increased g_s_ values by ~164% compared with Foph-inoculated plants without any elicitor treatment. In this sense, plants with three foliar BE, SA, and BR applications showed the highest g_s_ values (~184 mmol m^−2^ s^−1^), followed by Foph-inoculated plants with two SA (174.1 mmol m^−2^ s^−1^), BE (145.2 mmol m^−2^ s^−1^) and BR (144.6 mmol m^−2^ s^−1^) sprays (Figure 5A). JA sprays enhanced g_s_ values to a lesser extent in Foph-inoculated cape gooseberry plants.

**Figure 6:**
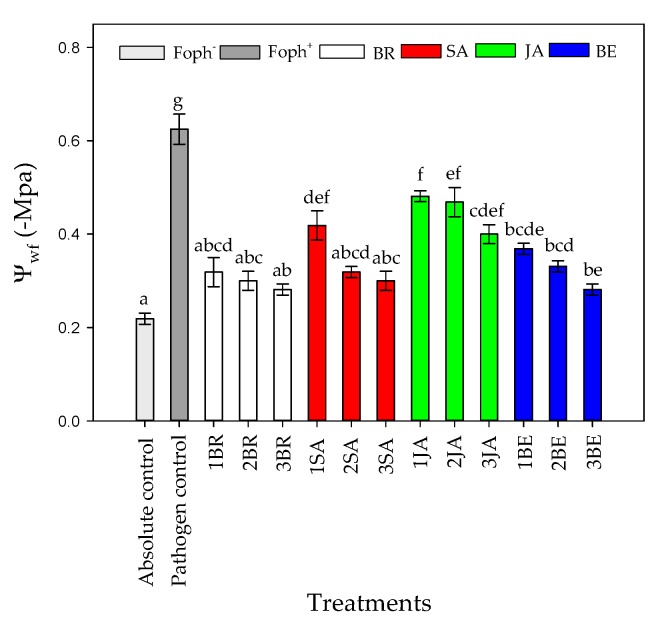
Effect of the different number of sprays (0, 1, 2, and 3 applications) of synthetic elicitors (Salicylic acid (SA), jasmonic acid (JA), Brassinosteroids (BR), or botanical extracts (BE)) on leaf water potential (Ψ_wf_) in *Fusarium oxysporum* f. sp. *physali* (Foph^+^) infected cape gooseberry (*Physalis peruviana* L.) plants at 50 DAI. Data represent the average of ten plants ± SE per treatment (*n* = 10). Bars followed by different letters indicate statistically significant differences according to the Tukey test (*p* ≤ 0.05).

Regarding T_L_, opposite results were found in comparison with g_s_ in which the higher T_L_ was recorded in Foph plants (pathogen control). Three foliar BR, SA, and BE sprays reduced T_L_ values in Foph-inoculated cape gooseberry plants (19.9, 20.3 and 20.6 °C, respectively) compared with inoculated plants without any synthetic elicitors (23.5 °C) (Figure 5B). On the other hand, foliar JA applications also generated a slight reduction in T_L_ values (21.6 °C) (Figure 5B). Finally, the most negative Ψ_wf_ values were recorded in plants only inoculated with Foph and without any foliar elicitor application (~−0.6 Mpa). Ψ_wf_ values were enhanced with the increase in the number of foliar elicitor sprays (mainly BR, SA or BE) (Figure 6). It was evidenced that the highest Ψ_wf_ values were also recorded in absolute control (−0.21 Mpa). Foliar JA applications in Foph-inoculated plants showed a slight increase in the Ψ_wf_ (Figure 6).

### 2.4. Parameters of Maximum Photochemical Efficiency of PSII (F_v_/F_m_) and Rapid Light-Response Curves

**Figure 7:**
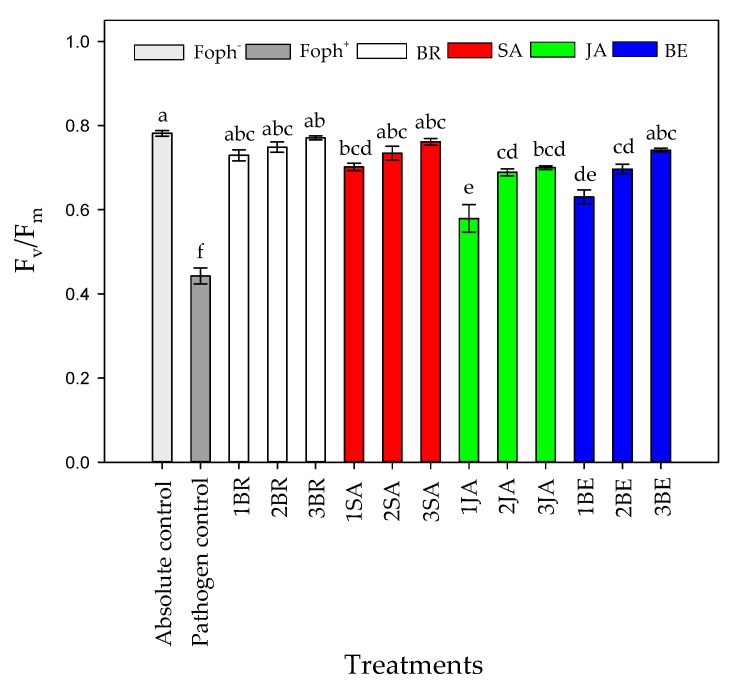
Effect of the different number of sprays (0, 1, 2 and 3 applications) of synthetic elicitors (Salicylic acid (SA), jasmonic acid (JA), Brassinosteroids (BR), or botanical extracts (BE)) on maximum photochemical efficiency of PSII (F_v_/F_m_) in *Fusarium oxysporum* f. sp. *physali* (Foph^+^)-infected cape gooseberry (*Physalis peruviana* L.) plants at 50 DAI. Data represent the average of ten plants ± SE per treatment (*n* = 10). Bars followed by different letters indicate statistically significant differences according to the Tukey test (*p* ≤ 0.05).

Significant differences (*p* = 0.000) were obtained between evaluated treatments (elicitors and number of applications) on F_v_/F_m_ ratio at 50 DAI. It was recorded that Foph-inoculated plants with or without any application of synthetic elicitors also recorded the lowest F_v_/F_m_ values (~0.44), while an increase of this ratio was obtained with a greater number of foliar elicitor sprays. In general, three foliar BR, SA, JA and BE applications caused a positive effect on the PSII photochemical efficiency, obtaining values between 0.7 and 0.77 (similar values were observed in absolute control) (Figure 7).

Differences were also obtained on the parameters derived from the rapid light-response curves (α (*p* = 0.000), ETR_max_ (*p* = 0.000) and I_k_ (*p* = 0.000)) at the end of the experiment. A linear trend (*p ≤* 0.001) was observed on α and ETR_max_ in the interaction between inoculation and synthetic elicitors application in which these two parameters were generally favored by the increase in the number of foliar elicitor sprays (Table 1). Finally, a directly proportional relationship was shown between I_k_ and the number of foliar applications of each synthetic elicitor. In this regard, I_k_ showed a quadratic trend with the use of BR (*p ≤* 0.001) and SA (*p ≤* 0.01), obtaining a higher reading with three foliar applications. Meanwhile, JA and BE presented a linear trend on I_k_ (*p ≤* 0.05 and *p ≤* 0.001, respectively) (Table 1).

### 2.5. Leaf Photosynthetic Pigments

**Figure 8:**
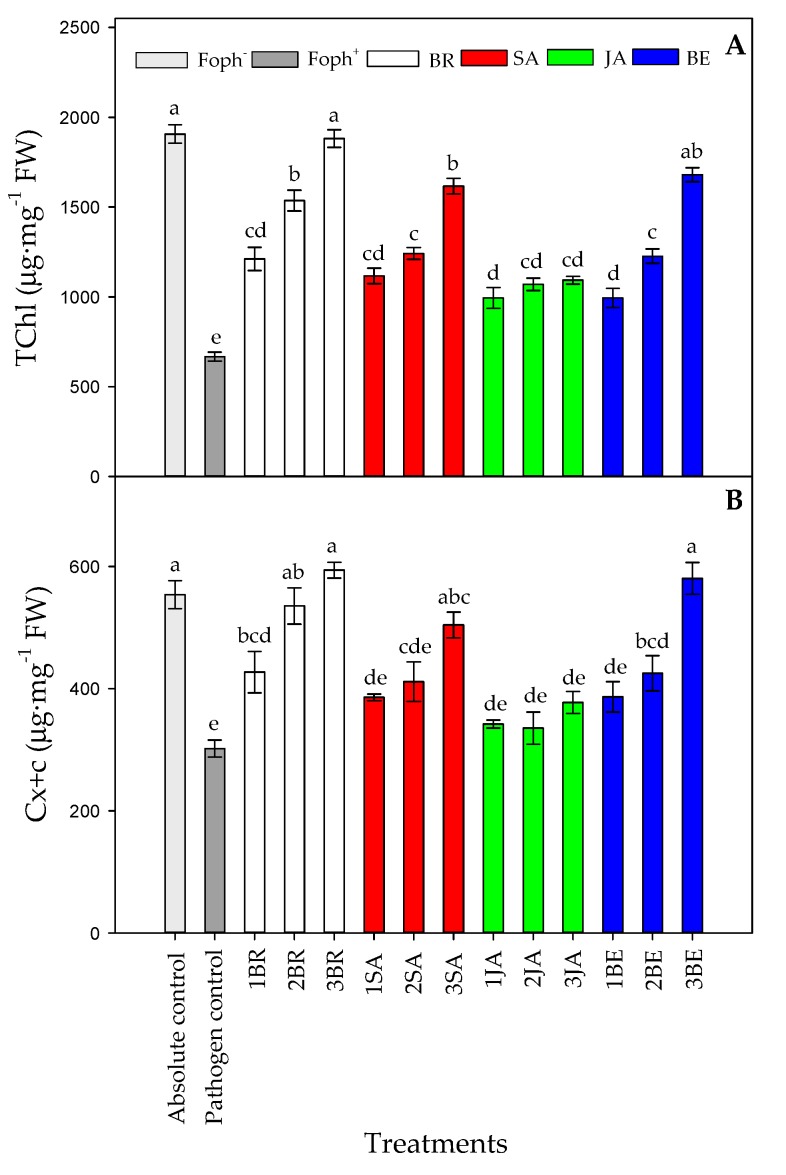
Effect of the different number of sprays (0, 1, 2 and 3 applications) of synthetic elicitors (Salicylic acid (SA), jasmonic acid (JA), Brassinosteroids (BR), or botanical extracts (BE)) on leaf total chlorophyll content (**A**) and leaf carotenoid contents (**B**) in *Fusarium oxysporum* f. sp. *physali* (Foph^+^) infected cape gooseberry (*Physalis peruviana* L.) plants at 50 DAI. Data represent the average of ten plants ±SE per treatment (*n* = 10). Bars followed by different letters indicate statistically significant differences according to the Tukey test (*p* ≤ 0.05).

Figure 8 summarizes the leaf photosynthetic pigments content (total chlorophyll (TChl) and carotenoids (Cx+c) content). Significant differences (*p* = 0.000) were also found on the leaf photosynthetic pigments between treatments (elicitors and sprays number) at the end of the experiment. In general, leaf photosynthetic pigments also increased with the number of foliar synthetic elicitor applications in Foph-inoculated plants (Figure 8). In this sense, it was observed that leaf photosynthetic pigment contents were mainly favored by three foliar BR (1881.8 mg g^−1^ FW for TChl and 594.1 mg g^−1^ FW for Cx+c), BE (1679.7 mg g^−1^ FW for TChl and 580.6 mg g^−v1^ FW for Cx+c), or SA (1616.7 mg g^−1^ FW for TChl and 504.3 mg g^−1^ FW for Cx+c) sprays in Foph-inoculated cape gooseberry plants, observing similar leaf photosynthetic contents obtained in absolute control plants (1831.5 mg g^−1^ FW for TChl and 521.4 mg g^−1^ FW for Cx+c). Finally, the lowest TChl and Cx+c was found in pathogen control (Foph plants) (Figure 8A,B).

### 2.6. MDA Production and Leaf Proline Content

**Figure 9:**
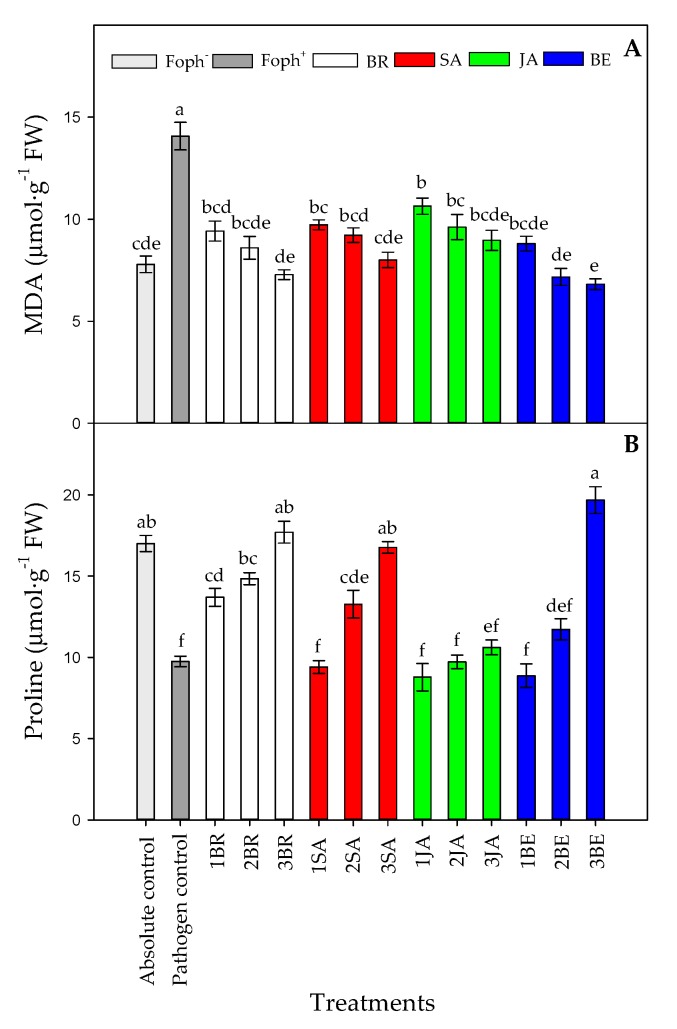
Effect of the different number of sprays (0, 1, 2 and 3 applications) of synthetic elicitors (Salicylic acid (SA), jasmonic acid (JA), Brassinosteroids (BR), or botanical extracts (BE)) on malondialdehyde (MDA) (**A**) and proline content (**B**) in *Fusarium oxysporum* f. sp. *physali* (Foph^+^) infected cape gooseberry (*Physalis peruviana* L.) plants at 50 DAI. Data represent the average of ten plants ± SE per treatment (*n* = 10). Bars followed by different letters indicate statistically significant differences according to the Tukey test (*p* ≤ 0.05).

MDA production (*p* = 0.000) and proline content (*p* = 0.000) were also affected by the treatments at 50 DAI. A lower lipid peroxidation was observed with the increase in foliar BE, BR or SA applications, obtaining similar values observed in uninfected plants (absolute control) (Figure 9A). On the other hand, a greater proline synthesis was mainly observed in Foph-inoculated cape gooseberry plants and treated with three foliar BR, SA or BE sprays. However, foliar JA applications did not cause any noticeable effect on proline production (Figure 9B).

### 2.7. Biplot Analysis of Physiological and Biochemical Responses to Foph Management with Synthetic Elicitors

**Figure 10:**
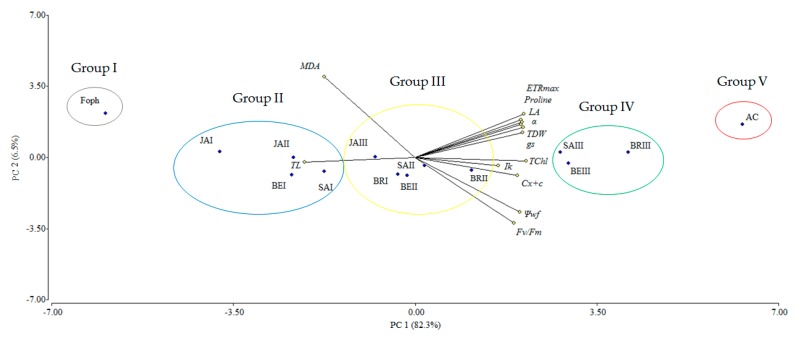
Effect of the different number of sprays (0, 1, 2 and 3 applications) of synthetic elicitors (Salicylic acid (SA), jasmonic acid (JA), Brassinosteroids (BR), or botanical extracts (BE)) on the biplot analysis of cape gooseberry plants inoculated with *Fusarium oxysporum* f. sp. *physali* at 50 days after inoculation (DAI). Abbreviations: AC, absolute control (Non-inoculated plants and without any synthetic elicitor spray); Foph, plants inoculated with only Foph without any synthetic elicitor spray; BRI, Foph-inoculated plants with one foliar BR application; BRII, Foph-inoculated plants with two foliar BR applications; BRIII, Foph-inoculated plants with three foliar BR applications; SAI, Foph-inoculated plants with one foliar SA application; SAII, Foph-inoculated plants with two foliar SA applications; SAIII, Foph-inoculated plants with three foliar SA applications; JAI, Foph-inoculated plants with one foliar JA application; JAII, Foph-inoculated plants with two foliar JA applications; JAIII, Foph-inoculated plants with three foliar JA applications; BEI, Foph-inoculated plants with one foliar BE application; BEII, Foph-inoculated plants with two foliar BE applications; BEIII, Foph-inoculated plants with three foliar BE applications; PC, principal component.

**Figure 11:**
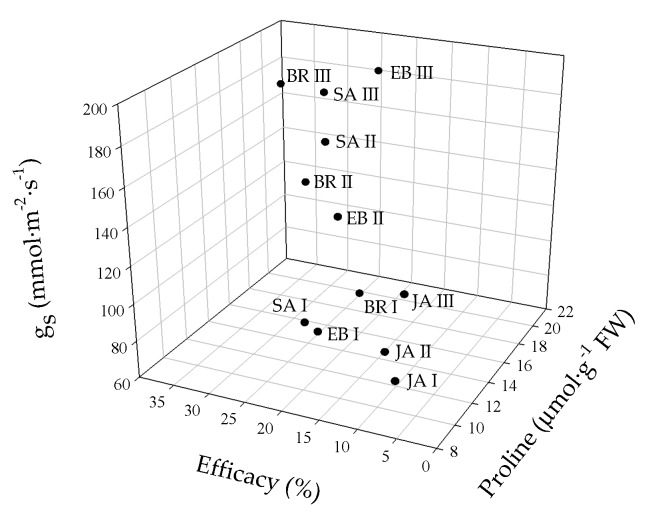
Three-dimensional plot (percentage efficacy, proline, and g_s_) for cape gooseberry plants inoculated with *Fusarium oxysporum* f. sp. *physali* (Foph^+^) and foliar applications with synthetic elicitors of resistance (brassinosteroids (BR), salicylic acid (SA), jasmonic acid (JA) or botanical extracts (BE)) at 50 days after inoculation (DAI). The data represent the mean of five data points. Abbreviations: BRI, Foph-inoculated plants with one foliar BR application; BRII, Foph-inoculated plants with two foliar BR applications; BRIII, Foph-inoculated plants with three foliar BR applications; SAI, Foph-inoculated plants with one foliar SA application; SAII, Foph-inoculated plants with two foliar SA applications; SAIII, Foph-inoculated plants with three foliar SA applications; JAI, Foph-inoculated plants with one foliar JA application; JAII, Foph-inoculated plants with two foliar JA applications; JAIII, Foph-inoculated plants with three foliar JA applications; BEI, Foph-inoculated plants with one foliar BE application; BEII, Foph-inoculated plants with two foliar BE applications; BEIII, Foph-inoculated plants with three foliar BE applications.

The principal component analysis (PCA) showed that the variables evaluated at 50 DAI explained 88.8% of the physiological and biochemical responses of Foph-inoculated cape gooseberry plants and treated with synthetic elicitors sprays (Figure 10). The variables are represented by vectors, while the elicitors and sprays number are identified by points. The vectors of g_s_, T_L_, Ψ_wf_, growth (TDW and LA), fluorescence parameters (F_v_/F_m_, α, ETR_max_ and I_k_) and biochemical readings (TChl, Cx+c, MDA and proline) have angles close to the origin, showing that there is a high correlation between plant physiological behavior and these variables. It was observed that non-Foph-inoculated plants and without any elicitor sprays (control plants) form a single group (V). In contrast, Foph-inoculated plants and untreated (No foliar elicitor applications) (group I) are located in the sector opposite to group V, showing a negative effect of Foph presence on plant physiological and biochemical responses. On the other hand, three differential effects were observed due to elicitor sprays: (i) Foph-inoculated plants and sprayed with JA (one and two applications) or one application of SA or BE (group II) behaved similarly to group I; (ii) three applications of JA, two applications of SA or BE and one application of BR (group III) had a minor positive effect on plant physiology in inoculated plants, whereas (iii) Foph-inoculated plants with three BR, SA or BE sprays (group IV) presented the best physiological and biochemical response showing a behavior close to group II (Figure 10). The three-dimensional plot (percentage efficacy, proline, and g_s_) corroborated the bi-plot analysis in which the correlation between physiological, agronomic, and biochemical variables showed that three applications of synthetic elicitors (BR, SA or BE) may ameliorate the disease level. These may be considered both responses against pathogens or stress mitigation via physiological processes (Figure 11).

## 3. Discussion

The effect of elicitors on the management of plant diseases may involve complex metabolic and molecular processes [13]. However, the objective of this study was to evaluate options with a practical application from the agronomic and physiological points of view. Studies to find complementary alternatives to manage vascular wilt caused by Foph in Andean fruit crops have been limited to the use of fungicides and have been recently focused on biological control agents [7,8]. Therefore, our research demonstrated the potential use of BR, SA or BE for Foph control in cape gooseberry. Three sprays of these synthetic elicitors decreased the progress of vascular wilt caused by Foph, as evidenced by the significant reduction of the AUDPC, vascular wilt index and vascular browning. Studies performed by Mandal et al. [17] and Rongai et al. [55] showed that a lower vascular wilt severity caused by *F. oxysporum* f.sp. *lycopersici* was observed when SA or BE were used in tomato (*Solanum lycopersicum* L.) plants, respectively. Likewise, browning of the vascular tissue is strong evidence of fusarium wilt [56]. In this sense, studies have shown that vascular browning indicates is good indicator of plant pathogenicity [57] Finally, it has been reported thar foliar BR application reduced vascular wilt severity caused by *F. oxysporum* f. sp. *cucumerinum* in cucumber (*Cucumis sativus* L.) plants [21].

Plant resistance induction under abiotic and biotic stress conditions can be regulated by physiological, biochemical and molecular mechanisms [13,58]. Similarly, these plant resistance responses can be stimulated by exogenous synthetic elicitors application [59,60]. The present study showed that the foliar application of synthetic elicitors, mainly BR (1 mL L^−1^), SA (100 mg L^−1^) or BE (2.5 mL L^−1^), helped to mitigate the negative effects caused by Foph by favoring physiological and biochemical responses in cape gooseberry plants.

Three foliar applications of BR, SA or BE favored physiological variables such as g_s_, Ψ_wf_, growth (TDW and LA) and chlorophyll fluorescence parameters (F_v_/F_m_, ETR_max_, I_k_ and α) in Foph-inoculated cape gooseberry plants (Figure 4, Figure 5, Figure 6 and Figure 7; Table 1). Bibi et al. [61] observed that cotton (*Gossypium hirsutum* L.) plants infected with *Verticillium dahliae* and sprayed with BR showed better leaf gas exchange parameters (leaf photosynthesis and transpiration), photochemical efficiency of PSII and greater dry matter accumulation (aerial part and roots) compared with plants without BR application. On the other hand, the positive effect of foliar SA sprays (100 mg L^−1^) has also been reported for pathogens such as *Phytophthora infestans* Mont. De Barry in tomato (*Solanum lycopersicum* L.) plants, favoring plant height and dry matter accumulation compared with diseased and untreated plants with SA [62]. Finally, Parvu et al. [63] recorded that tulip (*Tulipa gesneriana* L.) plants inoculated with *Botrytis tulipae* and treated with botanical extracts with high phenolic compound concentrations increased leaf transpiration and stomatal conductance compared with plants only with inoculation.

A higher leaf chlorophyll content after exogenous BR application has been also reported by several authors under biotic stress by plant pathogens. For example, Bibi et al. [64] observed that leaf chlorophyll a and b and carotenoids values in cotton (*Gossypium hirsutum* L.) plants infected with *Verticillium dahliae* and sprayed with BR were higher in comparison to diseased plants without BR. Biochemical markers such as MDA production, proline content and leaf photosynthetic pigments concentration are widely used to assess the plant response to conditions of abiotic or biotic stress [65,66,67].

Ding et al. [21] also reported a decrease in MDA production in cucumber (*Cucumis sativus* L.) plants inoculated with *F. oxysporum* f. sp. *cucumerinum* after foliar BR application. In addition, foliar BR application significantly increased proline values in cotton (*Gossypium hirsutum* L.) plants inoculated with *Verticillium dahlia* [61]. On the other hand, Zehra et al. [68] also observed a positive effect on the content of leaf photosynthetic pigments and proline in tomato (*Solanum lycopersicum* L.) plants affected by *F. oxysporum* f. sp. *lycopersici* after foliar treatment with SA. Also, SA application caused a significant decrease of MDA production in broad bean (*Vicia faba* L.) plants infected with *Bean yellow mosaic virus* (BYMV) [69]. Finally, Naz et al. [70] reported that wheat (*Triticum aestivum* L.) plants infected with *Bipolaris sorokiniana* and treated with BE rich in phenolic compounds showed an increase in the total chlorophyll content and antioxidant activity compared with diseased plants without exogenous BE application.

In this study, it was found that BR mitigated the negative effects caused by Foph by improving water relations, stomatal opening, and biochemical parameters, and decreasing vascular browning. This positive response can be associated to the fact that BR participate in the osmotic adjustment, promoting the accumulation of soluble carbohydrates such as starch and sucrose [71]. Additionally, BR applications favor the induction of SAR through the expression of enzymes such as β-1,3-glucanases, chitinases and peroxidases [31,72]. In this investigation, BR enhanced chlorophyll fluorescence parameters (F_v_/F_m_, ETR_max_, α, I_k_) in Foph-inoculated cape gooseberry plants. BR could cause a better chlorophyll parameter under disease conditions, since this plant hormone has a beneficial effect on PSII reaction centers, improving energy capture [73,74]. Also, the positive effects of exogenous BR applications could be related to the induction of expression of proline synthesis genes that favor a higher proline content, ROS scavenging, and plant cell membrane stability [75].

SA applications also decreased the severity of vascular wilt in Foph-inoculated cape gooseberry plants, which could be due to the positive regulation of genes that are coded for pathogenesis-related (PR) proteins, programmed cell death and ROS induction that help the signaling of plant defense mechanisms [75,76,77,78]. Likewise, SA showed a promoter effect of plant growth (TDW and LA) probably due to the fact that this molecule induces cell division and the biosynthesis of organic compounds, and increases nutrients availability and mobility [79]. A better plant water status (expressed as leaf water potential) in Foph-inoculated plants is due to the fact that SA enhances the accumulation of osmotic compounds such as soluble sugars and proline [80]. In the present study, SA applications also favored the leaf TChl content, since this plant hormone inhibits the activity of the enzyme ACC synthase, preventing ethylene formation and chlorophyll degradation [81]. Ghasemzadeh and Jaafar [82] stated that SA can also increase the antioxidant enzymes activity (expressed as a low MDA production) and leaf proline content under stress conditions, which was also observed in this study. These same authors also reported that SA helps the PSII reaction centers stability and the structure of enzymes such as Rubisco structures under stress conditions. Also, it is known that SA may induce resistance to Tobacco mosaic virus in part by enhancing the antioxidant capacity of the host plant [83,84]. Finally, other studies have also corroborated the role of SA in the defense mechanisms of plants is associated with the increase of phenolic and flavonoid compounds, reducing the damage caused by lipid peroxidation [85].

Three BE sprays reduced the vascular wilt parameters because BE can have antifungal activity since these extracts are constituted of secondary metabolites such as alkaloids, flavonoids, terpenoids and phenolic compounds. These metabolites can work as precursors of structural polymers such as lignin, or serve as signaling molecules [38,86]. In addition, BE sprays had a stimulating effect on all the physiological parameters evaluated in Foph-infected cape gooseberry plants. It has been reported that BE are considered biostimulants which can improve growth and total photosynthesis causing a greater plant biomass [87,88]. Furthermore, BE can favor the plant primary metabolism, causing an increase of free amino acids, proteins, carbohydrates, photosynthetic pigments levels and enzymatic activity [89].

A lower control of vascular wilt was observed in Foph-infected cape gooseberry plants and treated with JA compared with other elicitors. However, opposite results have been observed with this molecule, in which exogenous application has caused a decrease in the progress of diseases such as charcoal rot caused by *Macrophomina phaseolina* in forage legume (*Medicago truncatula*) plants [90], *Alternaria porri* f. sp. *solani* in tomato (*Solanum lycopersicum* L.) [91] and *Botrytis cinerea* in grapes (*Vitis vinifera L.* × *Vitis labrusca* L.) [92,93]. Several authors have also reported that SA activates plant defense responses against hemibiotrophic pathogens such as *F. oxysporum* [94,95,96]. The lack of response in Foph-infected plants and sprayed with JA may be due to the fact that JA is generally more effective against necrotrophic pathogens [97,98].

## 4. Materials and Methods

### 4.1. General Growth Conditions

An experiment under greenhouse conditions was carried out at the Faculty of Agricultural Sciences of the Universidad Nacional de Colombia, Bogotá (4°35’56” N, 74°04’51” W) between June and October 2018. The environmental conditions during the experiment were: 25/20 °C day/night temperature, 60%–80% relative humidity and a natural photoperiod of 12 h (1500 μmol m^−2^ s^−1^ photosynthetically active radiation at noon). Two-month-old ecotype ’Colombia’ (*Physalis peruviana* L.) seedlings purchased from a local nursery were used. The methodology described by Leslie and Summerell [99] was used to index seedlings to rule out *Fusarium oxysporum* f. sp. *physali* (Foph) infection. Then, seedlings were transplanted in 2 L plastic pots, containing a soil-based substrate (clay loam) and rice husk (3:1 *v/v*). After plant transplantation, seedlings were daily watered with 50 mL of a nutrient solution prepared from a complete liquid fertilizer (Nutriponic^®^, Walco SA, Colombia) at a dose of 5 mL L^−1^ H_2_O until the end of the experiment. The nutrient solution concentration was: 2.08 mM Ca (NO_3_)_2_·4H_2_O, 1.99 mM MgSO_4_·7 H_2_O, 2.00 mM NH_4_H_2_PO_4_, 10.09 mM KNO_3_, 46.26 nM H_3_BO_3_, 0.45 nM Na_2_MoO_4_·2H_2_O, 0.32 nM CuSO_4_·5H_2_O, 9.19 nM MnCl_2_·4H_2_O, 0.76 nM ZnSO_4_·7H_2_O, and 19.75 nM FeSO_4_·H_2_O. Finally, the water volume used was obtained by means of the daily quantification of plant evapotranspiration needs using the gravimetric technique described by Hainaut et al. [100].

### 4.2. Plant Inoculation and Resistance Elicitors Application

The inoculum used in the present experiment was the Foph-Map5 strain supplied by the Biological Control Laboratory of the Corporación Colombiana de Investigación Agropecuaria (Agrosavia, Mosquera, Colombia). Young mycelium segments from the supplied strain were cultured in 50 mL of Potato Dextrose Broth (PDB; Difco^TM^, Becton Dickinson, Sparks, MD, USA) in 250 mL Erlenmeyer flasks. Then, segments were incubated for 7 days under constant agitation in an orbital shaker (Lab-Line, Melrose Park, IL, USA) at 125 rpm and 28 °C under dark conditions [8].

The methodology described by Park [101] was used to confirm the absence of the pathogen (Foph) in the soil used in the substrate mixture. Subsequently, the inoculation of cape gooseberry seedlings was carried out by the addition of a liquid suspension of 100 mL of Foph at a concentration of 1 × 10^6^ microconidia mL^−1^ for each 0.9 kg of substrate (soil + rice husk), guaranteeing a final concentration of 1 × 10^4^ microconidia g^−1^ substrate [7]. Finally, two conditions of presence of the pathogen in the substrate (with and without Foph) were obtained.

Seedlings established in inoculated and non-inoculated substrate were sprayed with salicylic acid (SA) at a dose of 100 mg L^−1^, jasmonic acid (JA) at a dose of 10 mL L^−1^, brassinosteroids (BR) at a dose of 1 mL L^−1^, or commercial elicitor based on botanical extracts (BE) (Loker^®^, Biolchim SpA, Medicina, Bologna, Italy) at a dose of 2.5 mL L^−1^. Synthetic elicitors concentrations were selected based on the available literature about their use on diseases caused by plant pathogens: SA [17,102], Br [21,22], and JA [19,103]. BE concentrations were determined following the manufacturer’s recommendations. Finally, Table 2 summarizes the characteristics of the elicitors used in this study.

The foliar applications of all the elicitors used were performed at different moments during the experiment: 1) one foliar spray with synthetic elicitors (8 days before plant inoculation (DBI)) (1 spray); 2) two foliar elicitor sprays (8 DBI and at plant transplantation) (2 sprays); 3) three foliar sprays (8 DBI, plant transplantation and 8 days after inoculation (DAI)) (3 sprays), and 4) plants not treated with any elicitors (0 sprays). Additionally, a group of plants without Foph inoculation and without any foliar applications was used as absolute control. A total of 17 treatment groups were obtained and placed in the greenhouse in a completely randomized design where each experimental unit (treatment) consisted of ten plants (repetitions).

In general, the foliar elicitor sprays were carried out between 07:00 and 09:00 h, using a 1.8 L manual spray pump (Royal Condor^®^ Garden, Colombia) with an application volume per plant of 20 mL H_2_O, on the upper and lower leaf surface. SA was previously dissolved in ethanol (10%) and completed with deionized water to obtain the concentration to be used. JA was dissolved in methanol and completed with deionized water. Finally, BR were also dissolved in deionized water to obtain the concentration to be evaluated. All foliar applications were performed with a Tween^®^ 20 (Merck, Darmstadt, Germany) surface tension adjuvant at 0.02 % (*v/v*). Finally, the experiment lasted 75 days.

### 4.3. Analysis of Vascular Wilt Development

Vascular wilt severity in cape gooseberry seedlings was recorded each three days from plant inoculation to the end of the trial (50 DAI). Disease severity was evaluated using a scale with six levels described by Moreno-Velandia [104], where 0 = asymptomatic plants; 1 = slight epinastic response and mild chlorosis of the lower third of the plant; 2 = epinastic response in between 30%–50% of the leaves and moderate chlorosis in mature leaves; 3 = epinastic response in between 60%–80% of the leaves and moderate chlorosis in the middle third; 4 = epinastic response in all the leaves of the plant, severe chlorosis and defoliation; 5 = wilting and severe defoliation, dead plant. The disease severity index was calculated at each evaluation time using Equation (1) described by Townsend and Heuberger [105]:

(1) Disease severity index=(∑(nv)/V)

where *n* is the level of infection according to the scale, *v* is the number of plants present in each level and *V* is the total number of evaluated plants.

Also, the area under the disease progress curve (AUDPC) was estimated in each treatment by the trapezoidal integration method [106,107] using Equation (2):

(2) $$\mathrm{AUDPC}\;=\;\left\{\overset{n-1}{\underset{i=1}{\sum }}\left[\left(y_{i}+y_{i+1}\right)/2\right]*\left(t_{i+1}-t_{i}\right)\right\}$$

where *n* is the number of evaluations, $y_{i}$ and $y_{i+1}$ are the values of the severity scale that were present at each moment of evaluation and $\left(t_{i+1}-t_{i}\right)$ is the interval of time between evaluations. The presence or absence of Foph in plants of the different inoculated and non-inoculated treatments was confirmed 50 DAI by planting explants from the base of the plant stem in PDA medium (Oxoid, Basingstoke, UK) and incubating them at 25 °C [99].

Finally, the vascular browning was evaluated at 50 DAI by cutting vertical sections at the stem base in each treatment. The percentage of vascular bundle browning was quantified using a scale of five levels described by Mandal et al. [17], where 1 = no vascular browning; 2 =1–25% vascular browning; 3 = 26–50% vascular browning; 4 = 51%–75% vascular browning; 5 = >75% vascular browning.

### 4.4. Physiological and Biochemical Variables

All physiological and biochemical variables were assessed at 50 DAI in order to see the effect of the evaluated factors.

#### 4.4.1. Stomatal Conductance and Leaf Water Status

Stomatal conductance (g_s_) was estimated in the third fully expanded leaf from the upper portion of the canopy using a portable porometer (SC-1, Decagon Devices Inc., Pullman, Washington, WA USA) between 0900 and 1500 hours in totally sunny days. Leaf water status was monitored by measuring the leaf water potential (Ψ_wf_) using a Schollander pressure chamber (PMS Instruments, Albany, OR, USA) on the same leaves used to measure g_s_. Leaf temperature (T_L_) was also determined using an infrared thermometer (Cole-Parmer No. 800-323-4340) in plants from all treatments.

#### 4.4.2. Fv/Fm Ratio and Rapid Light-Response Curves 

The F_v_/F_m_ ratio and the rapid light-response curves (RLC) were determined using a modulated fluorometer (MINI-PAM, Walz, Effeltrich, Germany). After g_s_ estimation, the same leaves were acclimated to the dark using lightweight leaf clips for at least 15 min before measurements were taken. The F_v_/F_m_ measurements were performed with a pulse of maximum light intensity of up to 2600 µmol m^−2^ s^−1^ on the surface of the leaf. The RLC were constructed by plotting the electron transport rate (ETR) compared with the increase in actinic irradiance (from 1 to 1795 µmol m^−2^ s^−1^) with intervals of 10 s between the irradiance levels. In addition, the parameters α (Initial slope), ETR_max_ (maximum ETR) and I_k_ (light saturation parameter) were estimated by the model described by Xu et al. [108].

#### 4.4.3. Growth Parameters

Plants were harvested and their organs were separately weighed. Then, all leaves from each plant in every treatment were photographed with a digital camera (D3300, Nikon, Thailand) and saved as TIFF (Tagged Image File Format) images. LA was calculated from the obtained digital images using Java image processing software (Image J; National Institute of Mental Health, Bethesda, MD). Finally, leaf (LDW), stem (SDW) and root (RDW) dry weights from each plant per treatment were obtained. 

#### 4.4.4. Leaf Photosynthetic Pigments

The equations described by Wellburn [109] were used to estimate chlorophyll and carotenoid content. Leaf tissue samples (0.03 g) from the middle part of the canopy were collected and homogenized in 3 mL of 80% acetone. Then, the samples were centrifuged (Model 420101, Becton Dickinson Primary Care Diagnostics, MD, USA) at 5000 rpm for 10 min to remove particles. The supernatant was diluted to a final volume of 6 ml by adding 80% acetone [110]. The chlorophyll content was determined at 663 and 646 nm, whereas carotenoids were estimated at 470 nm using a spectrophotometer (Spectronic BioMate 3 UV-vis Thermo, Madison, WI, USA).

#### 4.4.5. Lipid Peroxidation (malondialdehyde-MDA)

The thiobarbituric acid (TBA) method described by Hodges et al. [111] was used to estimate membrane lipid peroxidation (MDA). Approximately 0.3 g of leaf tissue were also homogenized in liquid nitrogen. The samples were centrifuged at 5000 rpm, and then the absorbances were measured at 440, 532 and 600 nm with the spectrophotometer. Finally, an extinction coefficient (157 M·mL^−1^) was used to obtain the MDA concentration.

#### 4.4.6. Proline Concentration

The proline content was determined for all treatments using the method described by Bates et al. [112]. Approximately 0.3 g of the same leaves collected for the determination of photosynthetic pigments were homogenized in liquid nitrogen and stored for further analysis. Ten mL of a 3% aqueous solution of sulfosalicylic acid were added to the stored samples, which were then filtered with Whatman paper (No. 2). Subsequently, 2 mL of this filtrate were reacted with 2 mL of ninhydrin acid and 2 mL of glacial acetic acid. The mixture was placed in a water bath at 90 °C for 1 h. The reaction was stopped by incubation on ice. The resulting solution was dissolved in 4 mL of toluene by shaking the test tubes vigorously using a vortex shaker and the absorbance readings were determined at 520 nm with the same spectrophotometer used in the quantification of photosynthetic pigments (Spectronic BioMate 3 UV-Vis, Thermo, Madison, WI, USA). Proline content was calculated using the fresh weight of the sample by means of a standard calibration curve (equation 3).

(3) $$\frac{\mu \mathrm{mol}\;\mathrm{Proline}}{\;\mathrm{fresh}\;\mathrm{plant}\;\mathrm{material}}=\frac{\left[\frac{\left(\frac{\mu g\;Proline}{mL}\;x\;mL\;Toluene\right)}{\frac{115.5\;\mu g}{\mu mol}}\right]\;}{\left[\frac{g\;sample}{5}\right]}$$

### 4.5. Experimental Design and Data Analysis

Data were analyzed by two experimental designs. A factorial design in which the first factor corresponded to foliar synthetic elicitor (SA, JA, BR and BE) sprays and the second factor was the number of foliar sprays per elicitor (0, 1, 2 and 3 sprays) was used to analysis severity AUDPC, severity index, vascular browning and rapid light response curves. Meanwhile, the physiological and biochemical variables were studied using a completely randomized design (CRD). Each treatment group consisted of 10 plants as repetitions. The percentage values were transformed using the arcsine function. A principal analysis of components was performed with InfoStat 2016 software (analytical software, Universidad Nacional de Córdoba, Argentina) to select the best combination of synthetic elicitors and number of foliar applications under plant infection with Foph. For all analyzes, *F*-protected data analysis was used and when significant differences were obtained (*p ≤* 0.05) in the analysis of variance (ANOVA), a Tukey post hoc test was used for the comparison of means. Data were analyzed using Statistix v 9.0 software (Analytical Software, Tallahassee, FL, USA). Figures and three-dimensional plot were performed using Sigmaplot software (version 12.0, Systat Software, San José, CA, USA).

## 5. Conclusions

In summary, this study showed that the use of synthetic elicitors such as BR, SA, or BE improved the physiological and biochemical response in Foph-infected cape gooseberry plants. In this sense, the effectiveness of three foliar BR, SA, or BE sprays was corroborated by three-dimensional plot and biplot analysis, in which it can be shown that stomatal conductance, proline synthesis, and efficacy percentage were accurate parameters to predict Foph control. Therefore, these elicitors can be considered as commercial tools for vascular wilt management, probably minimizing applications of chemical synthesis fungicides. Similarly, it was observed that the use of these elicitors has positive effects on plant physiology, favoring g_s_, Ψ_wf_, growth (expressed as total plant biomass and LA), PSII efficiency, leaf chlorophyll and proline content, and low lipid membrane peroxidation. However, additional field studies are needed to quantify the effect of synthetic elicitors sprays (BR, SA, or BE) on yield parameters and crop quality in infected plants. The results obtained suggest that exogenous BR, SA, or BE applications in cape gooseberry plants could be considered within integrated management programs of vascular wilt in producing areas.

## Figures and Tables

**Table 1:** Effect of the interaction between synthetic elicitors (Salicylic acid (SA), jasmonic acid (JA), Brassinosteroids (BR), or botanical extracts (BE)) and the sprays number (0, 1, 2 and 3 applications) on the rapid light-response curve parameters (initial slope of the curve (α), maximum electron transport rate (ETR_max_) and minimum saturation irradiance (I_k_)) in *Fusarium oxysporum* f. sp. *physali* infected cape gooseberry (*Physalis peruviana* L.) plants at 50 DAI.

Treatments		Rapid Light-Response Curve Parameters		
Elicitor	Spray number	α	ETR_max_	I_k_
		µmol∙m^−2^∙s^−1^		µmol∙m^−2^∙s^−1^
			1	
BR	0	0.36 ^1^	4.21	10.27
	1	0.44	6.21	14.16
	2	0.59	7.09	11.85
	3	0.82	10.81	12.82
Significance ^2^		L, Q ***	L ***	Q, C ***
SA	0	0.36	4.16	10.32
	1	0.44	6.04	13.68
	2	0.57	7.47	13.07
	3	0.80	10.01	12.69
Significance		L ***	L ***	Q **
JA	0	0.37	4.19	10.25
	1	0.42	4.37	10.03
	2	0.55	5.92	10.79
	3	0.63	7.12	11.24
Significance		L ***	L ***	L *
BE	0	0.36	4.24	10.32
	1	0.43	5.29	11.82
	2	0.59	7.05	11.99
	3	0.75	9.39	12.43
Significance		L ***	L ***	L ***
CV ^3^ (%)		5.7	5.83	6.89

^1^ Data represent the average of ten plants per treatment (*n* = 10). ^2^ *, ** and ***: differ significantly in 0.05, 0.01 and 0.001, respectively. L = linear, Q = quadratic and C = cubic. ^3^ Coefficient of variation.

**Table 2:** Chemical name of the active ingredient, commercial name, and manufacturer of the synthetic resistance elicitors used to evaluate the foliar sprays on cape gooseberry plants inoculated with *Fusarium oxysporum* f. sp. *physali* (Foph).

Chemical Name of the Active Ingredient	Commercial Name (Manufacturer)
2-Hydroxybenzoic acid	Salicylic acid (Panreac Applichem, Barcelona, Spain)
(±)-1α, 2β-3-Oxo-2-(cis-2 pentenyl) Cyclopentylacetic acid	(±)- Jasmonic acid (Sigma Aldrich, MO, US)
(25 R) – 3β. 5α – dihydroxy-spirostan-6-one	Biomex DI-31 (Minerales exclusivos SA, Bogotá, Colombia)
Echinacea, Tormentil and Aloe extracts, K and Mg salts	Loker^®^ (Biolchim S. p. A., Medicina, Bologna, Italy)
